# An ethnoveterinary study on medicinal plants used by the Bai people in Yunlong County northwest Yunnan, China

**DOI:** 10.1186/s13002-023-00633-0

**Published:** 2024-01-13

**Authors:** Hongli Gao, Wei Huang, Chunyan Zhao, Yong Xiong

**Affiliations:** 1 Key Laboratory of Chemistry in Ethnic Medicinal Resources, State Ethnic Affairs Commission and Ministry of Education, School of Ethnic Medicine, Yunnan Minzu University, Kunming, 650504 People’s Republic of China; 2 Kunming Edible Fungi Institute of All China Federation of Supply and Marketing Cooperatives, Kunming, 650500 People’s Republic of China

**Keywords:** Ethnobotany, Bai people, Ethnoveterinary medicine, Yunlong County

## Abstract

**Background:**

The Bai people in Yunlong County, northwest Yunnan, China, have used medicinal plants and traditional remedies for ethnoveterinary practices. The Bai have mastered ethnoveterinary therapeutic methods in livestock breeding since ancient times. The Bai’s traditional ethnoveterinary knowledge is now facing extinction, and their unique ethnoveterinary practices have rarely been recorded. This study documented animal diseases, EMPs, and related traditional knowledge in Yunlong County, China.

**Methods:**

Ethnobotanical fieldwork was conducted in six villages and townships of Yunlong County between 2021 and 2022. Data were obtained through semi-structured interviews, participatory observations, and keyperson interviews. A total of 68 informants were interviewed, and the informant consensus factor and use reports (URs) were used to evaluate the current ethnoveterinary practices among the local communities. Information on livestock diseases, medicinal plants, and traditional ethnoveterinary medicine knowledge were also obtained.

**Results:**

A total of 90 plant species belong to 51 families, 84 genera were recorded as being used as EMPs by the Bai people, and Asteraceae plants are most frequently used. A total of 68 informants were interviewed, including 58 men (85.3%) and 10 women (14.7%). The most commonly used EMPs parts included the roots, whole plants, leaves, and stems, and the common livestock diseases identified in this field investigation included trauma and fracture, gastrointestinal disorders, respiratory disorders, parasitic diseases, miscellaneous, venomous snake bites, reproductive diseases, infectious diseases, skin disease, and urinary diseases. Most of the EMPs are herbs (77.78%). Courtyard is one of the habitats of medicinal plants in Yunlong County.

**Conclusion:**

Traditional knowledge of ethnoveterinary medicine is related to the local sociocultural characteristics of the Bai. Plants are used in cultural traditions, which, in turn, nourish the plant culture. Cultural diversity and biodiversity are interdependent. This traditional knowledge is at risk of disappearance because of the increasing extension of Western veterinary medicine, lifestyle changes, and mainstream cultural influences. Therefore, it is important to continue research on ethnoveterinary practices.

**Supplementary Information:**

The online version contains supplementary material available at 10.1186/s13002-023-00633-0.

## Background

Medicinal plants refer to plants that have been recognized and utilized by people to treat human and animal diseases. Medicinal plants play a role in protecting the lives and health of ethnic groups living in the remote areas of developing countries [1, 2]. Some of these practices have also been applied in developed areas [3, 4]. At least 80% of developing countries rely mainly on local traditional medicine to prevent and treat various diseases in humans and animals [5]. Medicinal plants are an important basis for the emergence and development of Chinese medicine [6–8]. Ethnoveterinary medicines are generally defined as those used based on folk expertise, beliefs, knowledge, practices, methods related to animals’ health, and to cure various ailments in the ethnic group areas [9]. Ethnoveterinary medicine is not only an important part of traditional medicine but also an indispensable part of local animal health and the most basic veterinary services [10, 11].

Ethnoveterinary medicine plants (EMPs) are the plants used to prevent and control animal diseases, especially in remote and undeveloped areas where access to medical care is limited or missing. EMPs have a long history of practice, especially in countries with more developed animal husbandry practices [12–17]. At the last ten years, the topic of ethnoveterinary has developed a great interest among researchers in China, such as many surveys of ethnoveterinary and EMPs [18, 19]. Traditional low-cost methods for treating animal diseases, rather than synthetic drugs, are often desired.

The Bai people are the ancestral home of Yunnan [20]. With a population of 2.09 million, the Bai ethnic group is the 15th largest in China [21], mainly distributed in Yunnan, Guizhou, Hunan, and other provinces [22]. Most of the Bai people in China reside in the Dali Bai Autonomous Prefecture of Yunnan Province. The Dali Prefecture was the origin and main settler of the Bai people. The Bai nationality has its own language and belongs to the Bai branch of the Tibetan-Burmese family of the Sino-Tibetan language family [20]. Based on the regional and national characteristics,  Bai and Chinese bilingual bicultural education are carried out for Bai students. Buddhism and “Benzhu” worship constitute an important part of the Bai religious culture. “Benzhu” worship is a unique religious belief of the Bai nationality, which is generally a hero in local myths and legends, and the Bai people regard “Benzhu” as the local protection god [23].

Bai medicine has a long history, and archaeology has been used since the Ming Dynasty. Bai ancestors generally used local herbs or traditional Chinese medicines to treat diseases [24]. Bai medicine is an accumulation and summary of the experiences of the Bai people in disease prevention and treatment over generations. Its diagnosis and treatment are characteristic of “Medicine with God.” Treating diseases and praying to gods do not conflict with each other. “Medicine with God” means doctors praying in the healing process. The generation of “Medicine with the God” is bound to primitive religious, cultural, and economic factors [25].

Local people have developed special diagnostic and treatment methods, such as spa, moxibustion, rolling egg (roll a shelled, boiled egg on the area of discomfort for relief), steam, and medicinal dietary therapies, and these therapies integrate the medical theories and methods of Han, Yi, Tibetan, and other ethnic groups. They were also good at using single-experience prescriptions [26]. Finally, a medical culture with national and regional characteristics was formed, which contributed greatly to the reproduction of the Bai people, including the research on the medicinal culture of Yunnan Bai people [27], Yunnan Bai people medicine [28], habitual plant medicine of the Bai people [29], and illustrated guide of medicinal plants of the Bai people [30]. In China, traditional knowledge of ethnoveterinary medicine originates from the daily livestock management of indigenous people and the long history of these practices.

The Yunlong County is located west of Dali Bai Autonomous Prefecture and is a collection of remote mountainous areas, ethnic groups, poor areas, and alpine areas [31]. There are five deeply poor townships in Dali Bai Autonomous Prefecture, and there are four in Yunlong County, accounting for 80% of the deeply poor townships in the prefecture. According to the poverty standard line of 2300 yuan (per capita annual net income) established in 2011, Yunlong County had a total of 151,900 poor people in that year (the total population was 207,117) [32]. It has a large population in both mountainous and semi-mountainous regions. The Bai people in Yunlong County have a long history of livestock and poultry farming. They are rice farmers with a long history of farming in the plateau region, although the rice-planting area is small in Yunlong County, and the local economy is mainly mountainous agriculture.

There are more than ten ethnic groups, including the Bai, Han, Yi, Miao, Hui, Dai, Lisu, and Achang, with the Bai people, accounting for the largest proportion (72.7%) of the total population [24]. Differences in culture, religion, customs, language, dietary habits, and living environments between ethnic groups have prompted the generation of several traditional medicines with distinct regional characteristics [33]. According to the results of the seventh national census, the population living in cities and towns in Yunlong County was 51,298, accounting for 28.04% of the total population. The population living in rural areas was 131,679, accounting for 71.96% of the total population [34].

**Fig. 1:**
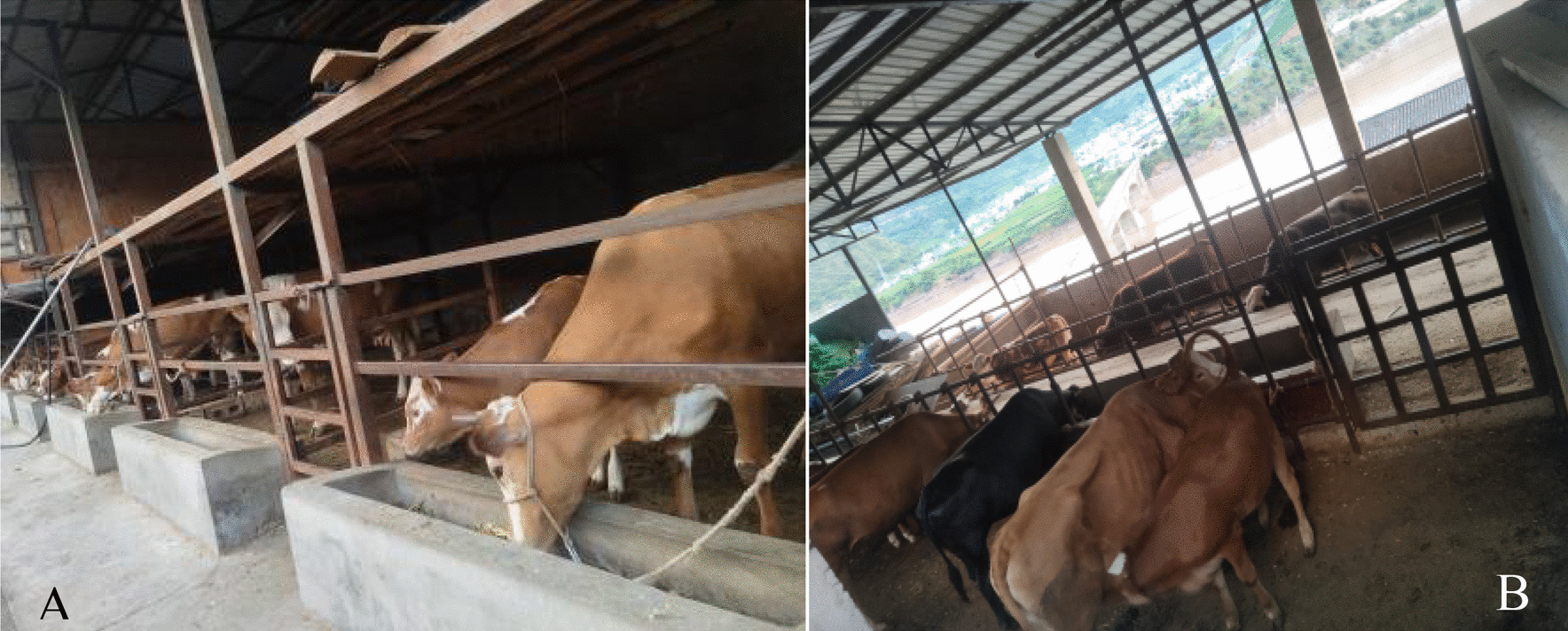
Tuanjie Township Biao Village Beef cattle Farm (**A**) and Miaowei Township breeding Farm (**B**), and two photographs were taken by Hongli Gao on August 9, 2021

Most of the rural population is scattered on mountain tops or hillsides; mountain roads are muddy and potholed, and transportation is inconvenient. These areas are far from urban areas, and agriculture and animal husbandry are the main sources of income. Indigenous people have a long history of raising livestock and poultry, such as black goats, pigs, cattle, donkeys, chickens, and ducks, to meet their needs and as a source of family income. In addition, according to the Circular on the 13th Five-Year Plan for Poverty Alleviation issued by the State Council on November 23, 2016 [35], The Yunlong County established several farms in various townships based on the advantages and endowments of natural resources to revitalize the countryside (Fig. 1).

The local agricultural department crossbred non-local beef cattle with local breeds to increase meat production. Additionally, it provided policy assistance and economic support to local residents. Veterinarians have used medicinal plants to treat animal diseases, forming a set of unique knowledge systems for local traditional veterinary medicine; however, to date, these have not been systematically studied. Detailed information on the use of traditional ethnoveterinary knowledge by the Bai people in Yunlong County is lacking. In this study, ethnobotanical methods were used to investigate and catalog traditional EMPs in Yunlong County, China. This investigation will contribute to the cataloging of medicinal plants for the treatment of livestock diseases and uncover relevant knowledge of traditional Bai medicine.

## Methods

### Study area

The Yunlong County, Dali Bai Autonomous Prefecture, is located in the western part of Yunnan Province, in the longitudinal valley of the Lancang River at the southern end of the Hengduan Mountain, between 98°52′–99°46′ E and 25°28′–26°23′ N. It is located at the junction of Dali Prefecture, Baoshan area, and Nujiang Prefecture, and the total area is 4400.95 km^2^, 90% of which is mountainous [36]. The distribution characteristics of the water systems in the territory are clear. The Lancang River and its tributaries run west and in the middle of Yunlong County from north to south, respectively. The riverbed has a large slope and is rich in hydraulic resources. The basic topography is high from east to west, low in the middle, gradually decreasing from north to south, and the elevation is approximately 2000–2500 m. The Yunlong County generally has a continental subtropical plateau monsoon climate with distinct dry and wet seasons, the same season of rain and heat, and the same period of dryness and cooling [37].

**Fig. 2:**
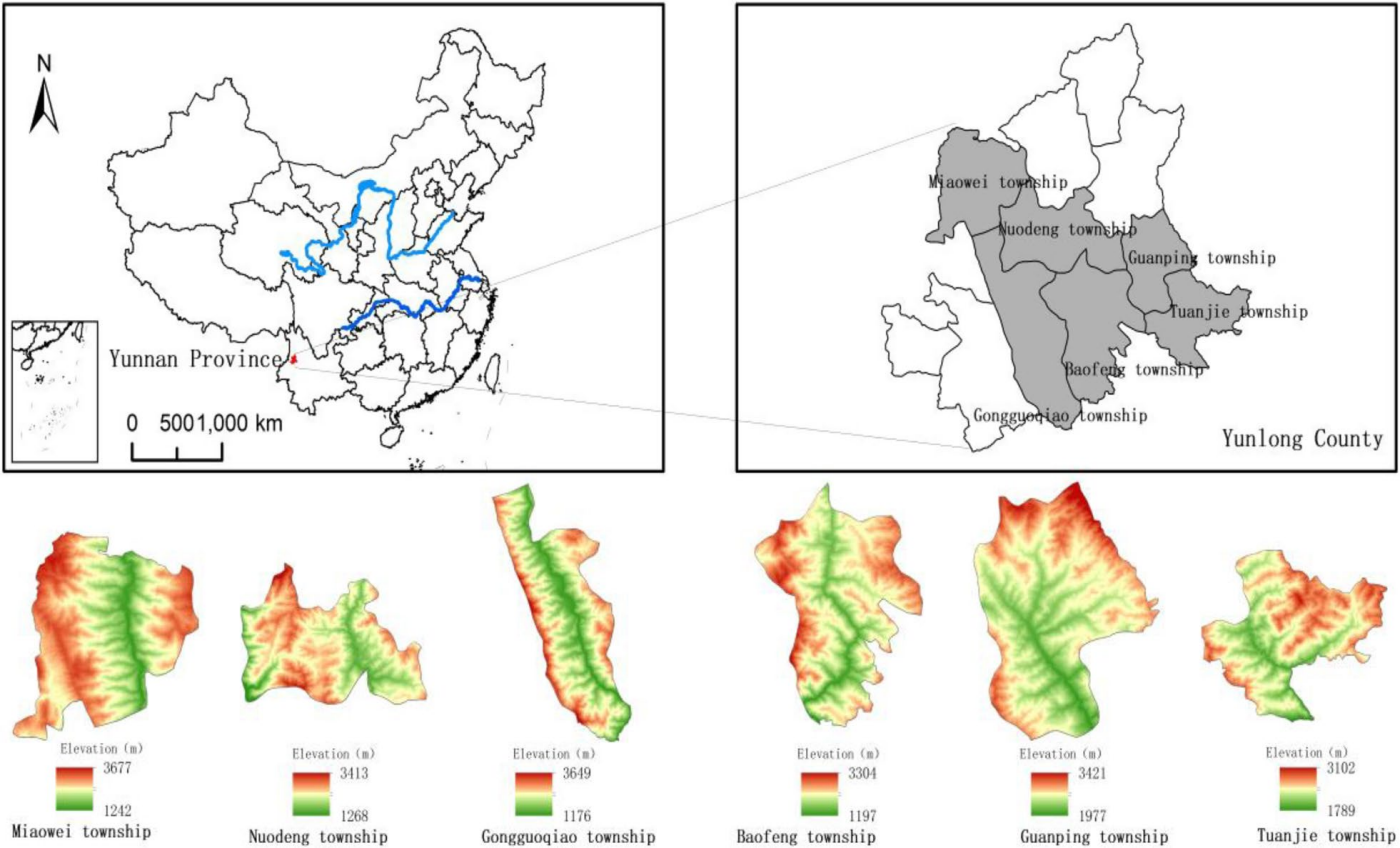
Location of the Yunlong County in China and elevation map of the townships in study area

It is a complex and changeable “compound three-dimensional climate.” The annual average temperature in Yunlong County is 16.1℃, the hottest monthly average temperature is 22.3℃, and the coldest monthly average temperature is 8.4℃. The difference between the annual average temperature at the highest and lowest elevations is 17℃ [38]. The local mountains are undulating, the forests are dense, the rivers are vertical and horizontal, the sunshine is sufficient, the rainfall is moderate, and the climate is suitable, which provides superior conditions for the growth and reproduction of all kinds of animals and plants, so it is rich in plant resources and is a natural medicinal resource bank. Yunlong County has jurisdiction over 11 townships, including Miaowei, Guanping, Baofeng, Nuodeng, Gongguoqiao, and Tuanjie. This survey area included six villages (Biaocun, Dalishu, Xiaomaidi, Nuodeng, Gongguoqiao, Tuanjie), three local herbal medicinal markets (Tenlong, Miaowei, Baofeng), three traditional animal breeding farms, and four herbal medicine planting bases (Yunlong County Yuanheng biotechnology development Co., Ltd, Songping, Longze, Yunlong county Canwen) from Yunlong County (Fig. 2).

### Data collection

**Fig. 3:**
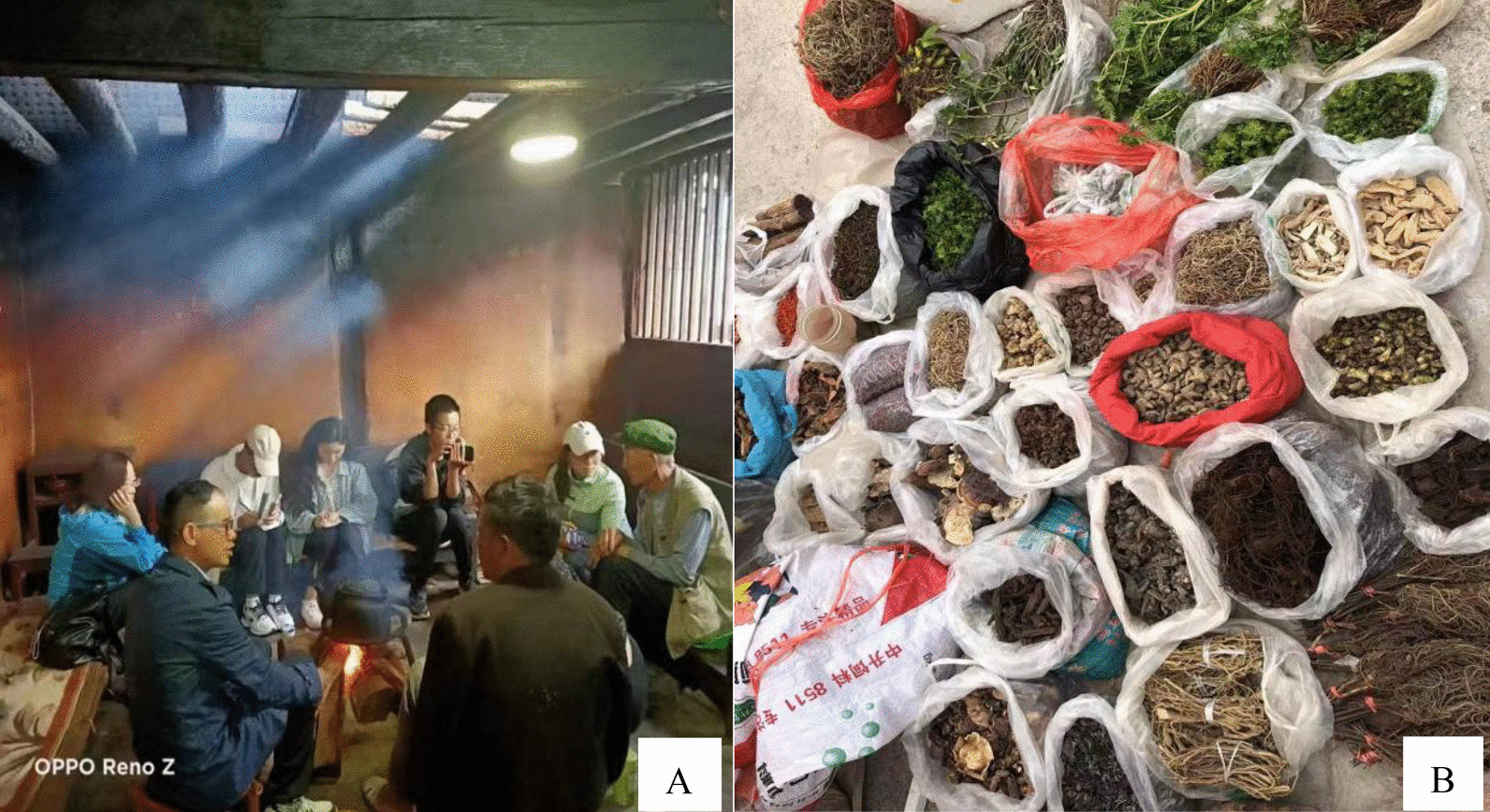
Key person interview (**A**) was taken by local residents on January 14, 2022, in the Guanping township xiao di pang Village; local herbal medicinal markets (**B**) were taken on August 8, 2022 in Baofeng, and two photographs were taken by Hongli Gao

**Fig. 4:**
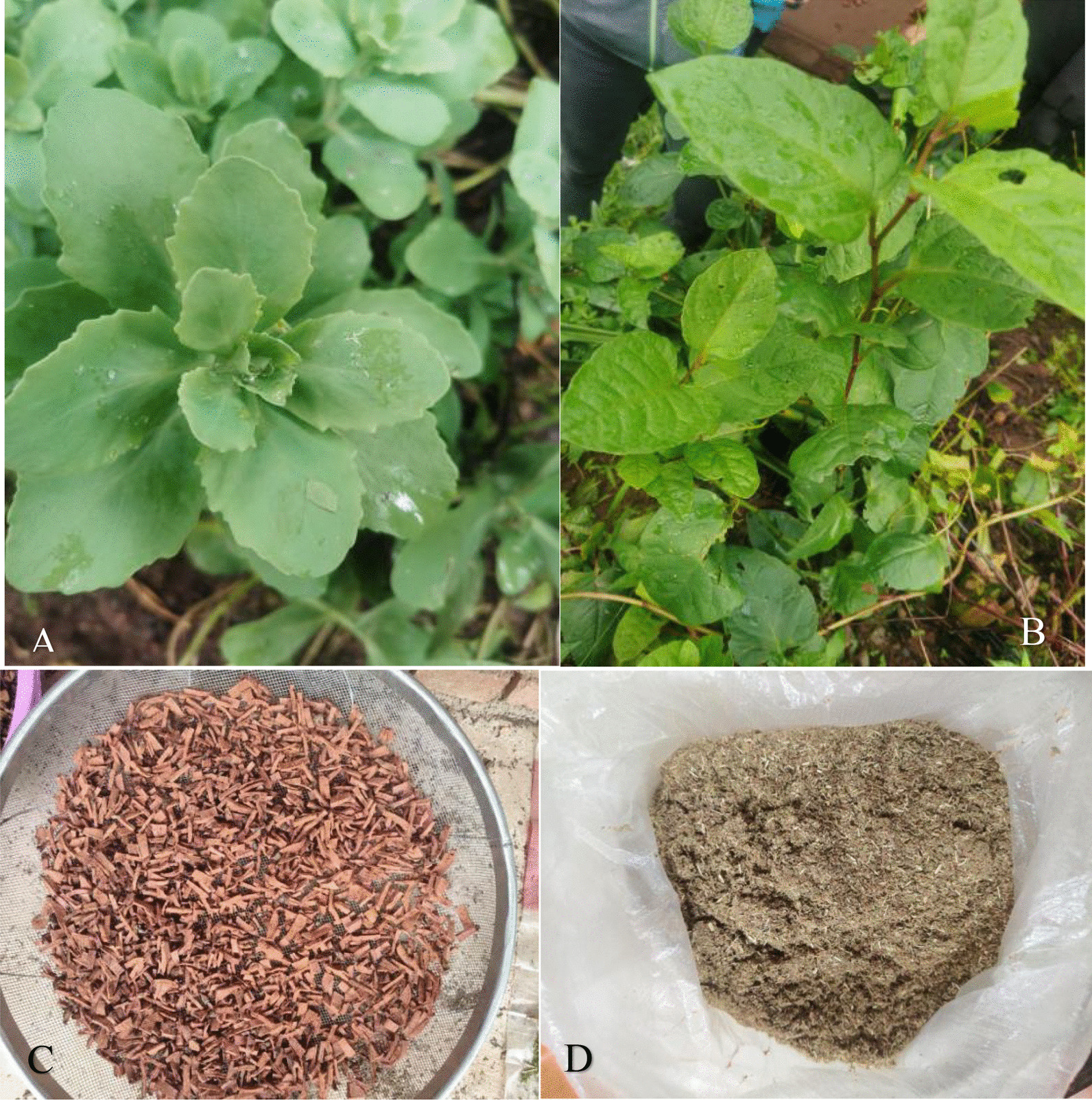
Fresh herbs (**A** *Phedimus aizoon* (L.) 't Hart and **B** *Reynoutria japonica* Houtt.), dried herbs (**C** *Berberis diaphana* Maxim.) and prepared herbs (**D** *Botrychium ternatum* (Thunb.) Sw. And *Lycopodium japonicum* Thunb.), and two photographs were taken by Hongli Gao in Baofeng on September 3, 2021

Ethnobotanical investigations were conducted from August 2021 to August 2022, which included structured interviews, participatory observations, semi-structured interviews, and key person interviews combined with field investigations to complete the cataloging of traditional EMPs in Yunlong County (Fig. 3). All interviews were conducted by Wei Huang in the local Bai language. A total of 68 local residents were interviewed, including 58 men and 10 women. These were farmers and herbal veterinarians with several years of experience in raising and treating livestock diseases. Local farmers with knowledge of veterinary medicine would collect some herbs (Fig. 4a, b) and hang them at home for drying on rainy days (Fig. 4c). Herbal veterinarians would prepare the medicine according to common local veterinary diseases and save it for later use (Fig. 4d).

The informants were apprised of our purpose before the interview to gain consent and trust so that we could communicate freely and openly with them. The primary content of the interviews consisted of “5W + H” questions (i.e., questions concerning what, when, where, who/whom, why, and how the informants utilized EMPs). The recorded information was shown again to the informant again to avoid errors and tampering.

Plant specimens were collected by the first author and identified using the “Flora of China” and China Digital Plant Museum (https://www.cvh.ac.cn/). The Latin names of the plants were corrected and verified using The Plant List (http://www.theplantlist.org/). The voucher specimens were stored in the plant specimen room of the Key Laboratory of Ethnic Medicine Resources Chemistry, Yunnan Minzu University, Kunming, China.

### Data analysis

For each plant collected, according to its use report (URs), the UR was defined as the type of disease treated by the plant [39]. To analyze the differences in medicinal plant species used by different herbalists in the treatment of a certain type of disease [40], the diseases reported by the interviewer were divided into 10 categories. The informant consensus factor (FIC) was calculated as follows:

$${\text{FIC}} = \left( {{\text{Nur}} - {\text{Nt}}} \right)/\left( {{\text{Nur}} - {1}} \right)$$

Nur represents the sum of the number of plant species used by all informants to treat a particular disease and Nt is the number of plant species commonly used by all informants to treat the disease. The FIC value ranges from 0 to 1, and the higher the FIC value, the higher the difference in plant species used to treat a disease; the lower the FIC value, the more concentrated the plant species used in the treatment of disease [40].

## Results

### Informant characteristics

A total of 68 informants were interviewed, including 58 men (85.3%) and 10 women (14.7%). The informants ranged in age from 30 to 79 years, with most being older than 50 years (50%), and the average age was 52 years (Table 1). These included farmers, herbalists, truck drivers, etc. They were localities with several years of experience treating livestock diseases. Most of them had low primary school education levels.

**Table 1:** Characteristics of informants

Characteristics	Frequency	Percentage (%)
*Sex*		
Male	58	85.3
Female	10	14.7
*Age range (y)*		
30–40	10	14.7
41–50	24	35.3
≥ 50	34	50
*Occupation*		
Farming	38	55.9
Truck driver	11	16.2
Veterinarian	15	22.1
Other	4	5.8

### Ethnoveterinary medicinal plant diversity

**Fig. 5:**
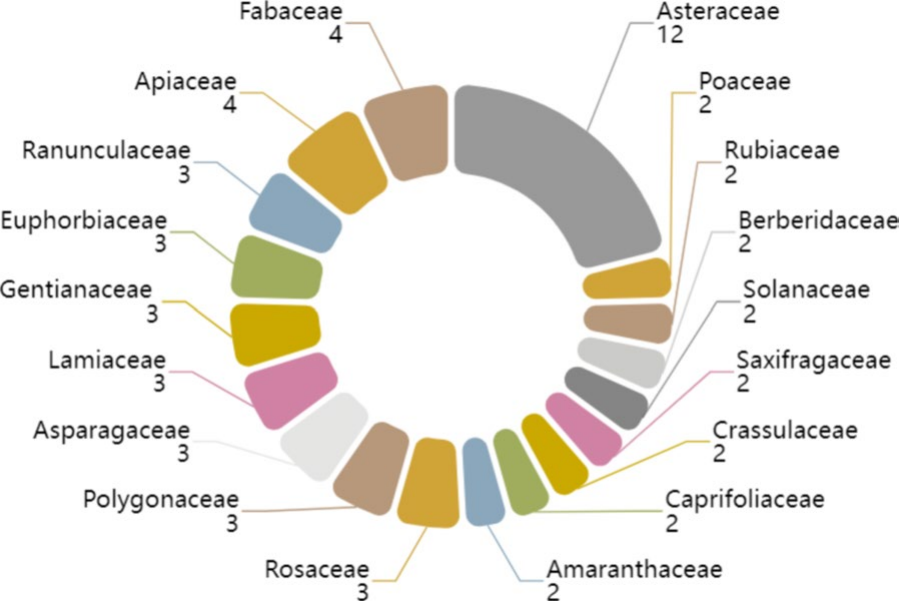
Distribution of plant species number on the basis on families

A total of 90 plant species belonging to 51 families and 84 genera were recorded. The Asteraceae (12 spp.) family had the highest number of individual species used in ethnoveterinary practices, followed by Fabaceae (4 spp.) and Apiaceae (4 spp.). Figure 5 shows the EMPs in the Bai region, 7 families had 3 species, 8 families had 2 species, and 33 families had only 1 species.

### Common livestock diseases in Yunlong County

**Fig. 6:**
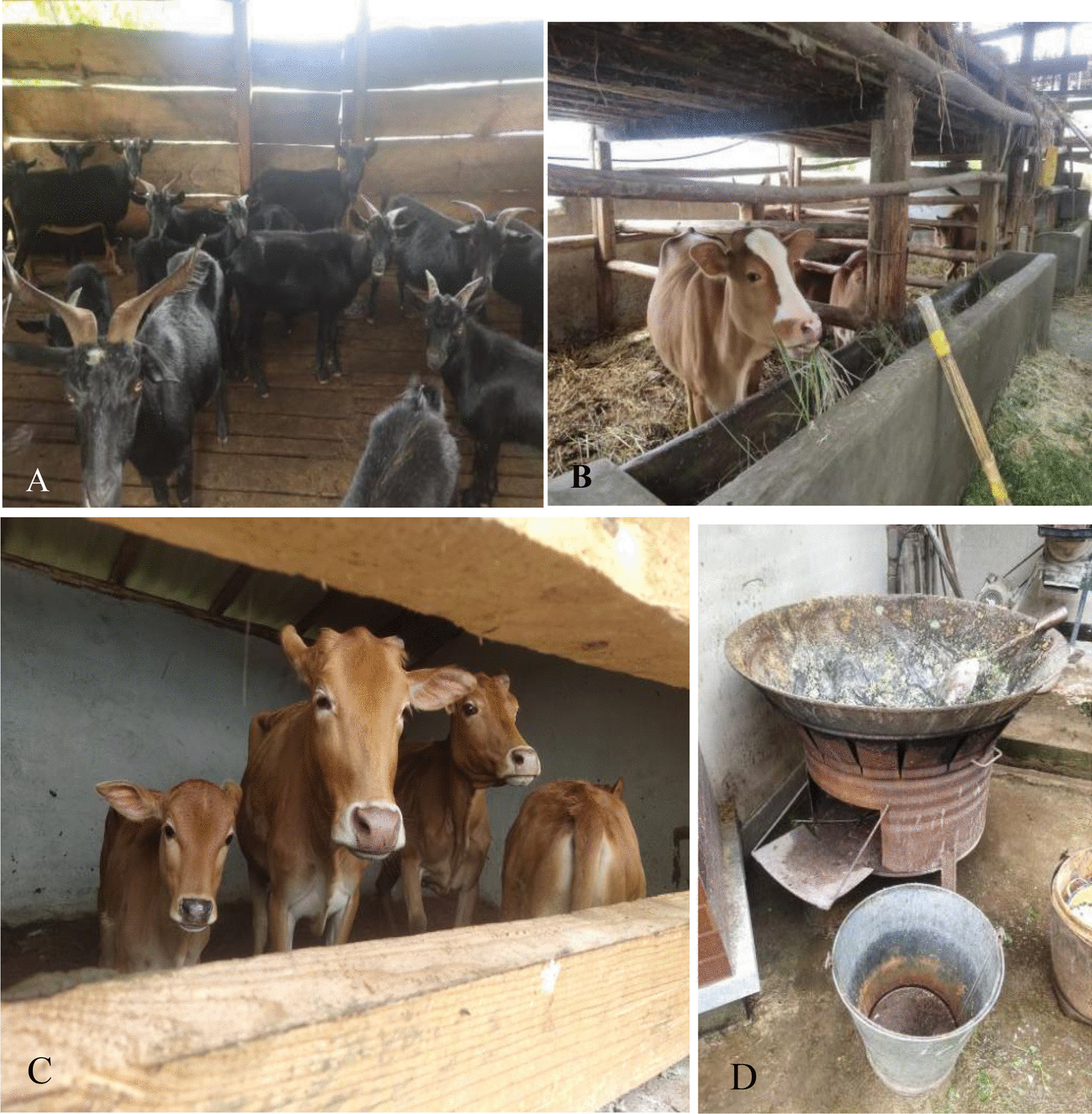
Local breeding livestock (**A**–**C**) and pig pots (**D**) were taken by Hongli Gao in Gongguoqiao on August 17, 2021

As can be seen from Table 2, fall injury is the most common disease of large livestock in Yunlong County, followed by gastrointestinal diseases, respiratory diseases, snake bites, and other diseases. The Yunlong County is characterized by high mountains and steep slopes. Local livestock mostly adopt modes of grazing and free-raising, which makes them prone to infectious diseases caused by traumatic wounds. Hot and humid climatic conditions may lead to wound inflammation and slow healing; moreover, gastrointestinal, respiratory, and parasitic diseases often occur in enclosures with poor sanitary conditions. In recent years, economic development has driven improvements in hygiene levels, and the importance of enclosures for healthy livestock growth is increasingly being recognized. In spite of its simplicity, a traditional breeding farm emphasizes regular cleaning of the enclosure. The sheepfold is usually designed with two layers, with a gap in the boards. Most of the feces fall through the gap to the lower layer, which helps maintain cleanliness of the pen while enabling easy cleaning (Fig. 6A). The use of dry straw washers not only protects cattle and other large livestock from falls and injuries but also provides optimal composting conditions and increases nutrients for crops when using the compost of manure mixed with straw (Fig. 6B, C). Many local farmers maintain the traditional habit of livestock feeding, which greatly reduces the possibility of parasitic infection and diseases (Fig. 6D). The Yunlong County has several retail investors, mostly living on hilltops or hillsides with lush vegetation in the front and back of houses. Livestock may be bitten by snakes during grazing and while in captivity. Reproductive diseases are common in patients with dystocia, persistent placenta, or postpartum weakness.

**Table 2:** Common livestock diseases in Yunlong County

Disease categories	Number of species	Citations	FIC
Trauma and fractures	32	54	0.42
Gastrointestinal disorders	18	50	0.65
Respiratory disorders	16	48	0.68
Parasitic diseases	15	40	0.64
Miscellaneous	14	60	0.78
Venomous snake bites	14	38	0.63
Reproductive diseases	10	42	0.78
Infectious diseases	8	50	0.85
Skin diseases	7	38	0.84
Urinary diseases	5	12	0.64

Foot-and-mouth disease is an occasional infectious disease, the control of which is mainly based on prevention, adhering to the principle of “early detection and early treatment.” Once discovered, the infected animals are immediately killed and buried. Foot-and-mouth disease occurs in many areas of China. According to the announcement of the Ministry of Agriculture and Rural Affairs of the People’s Republic of China, for the relevant livestock breeds throughout the country, according to the local actual situation, the appropriate immune vaccines against foot-and-mouth disease type O and A are selected on the basis of scientific evaluation, and the import of epidemic products from abroad is prohibited [41]. The interviewer was blinded to whether Radix Isatidis, Milkvetch Root, Palmatine, and other proprietary Chinese medicines were supplemented to livestock feed for preventing infectious diseases. The interviewers were also blinded to the specific types of diseases that were termed miscellaneous, including fever, nose bleeding, edema, loss of appetite, and abnormal conditions related to various organ systems of the animal. Miscellaneous information is related to the limitations of local residents’ miscellaneous medical knowledge.

### Life forms and parts of plants used for ethnoveterinary purposes

**Fig. 7:**
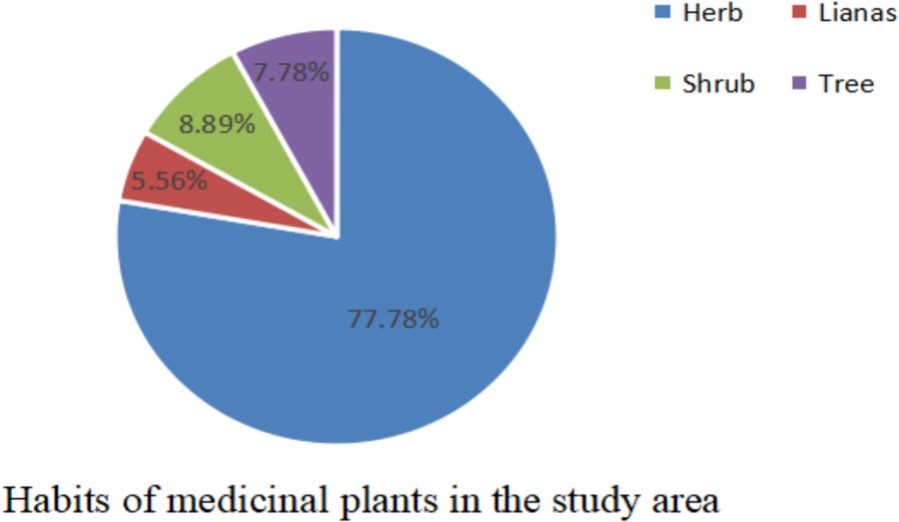
Ethnoveterinary medicinal plant life form

The survey found that among the 90 EMPs (Additinal file 1: Table 3), herbs accounted for the largest proportion with 70 species (77.78%), followed by eight shrub (8.89%), seven trees (7.78%), and five liana species (5.56%), as shown in Fig. 7. This distribution is closely related to local climatic conditions. The Yunlong County has a continental subtropical plateau monsoon climate with abundant shrubs, trees, and herbaceous plant types. Herbaceous plants have a short growth cycle and large growth, which are sufficient to meet the demand and are easy to harvest and process.

**Fig. 8:**
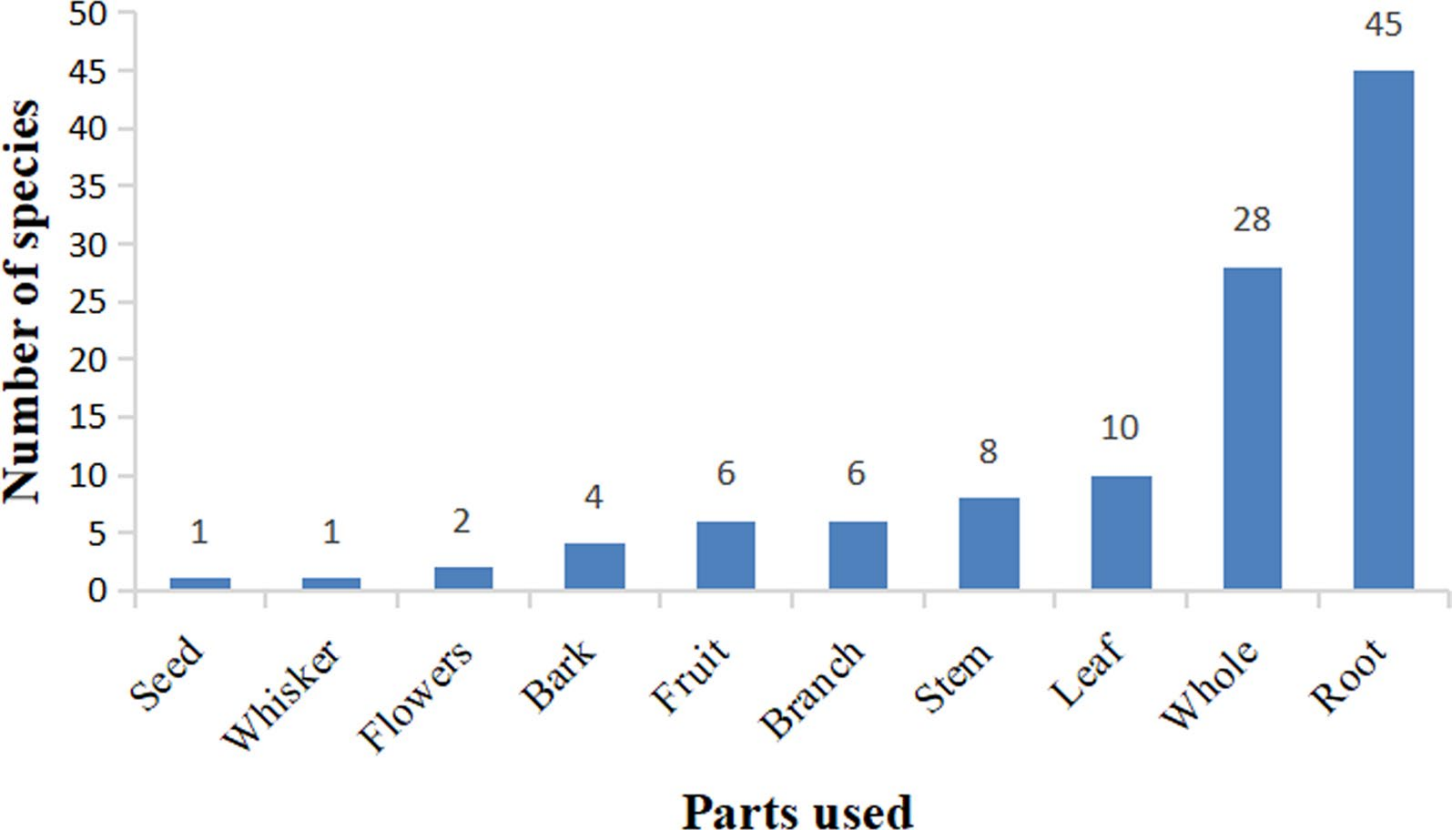
Distribution of plants used in the ethnoveterinary practice of the Bai people according to the frequency of plant parts

The total number of medicinal parts was 111 (some plants contained multiple medicinal parts). Roots were the most frequently used plant parts, constituting 40.54%, followed by whole plants (25.23%), leaves (9.01%), stems (7.20%), and mixed plant parts (18.02%) (Fig. 8).

Root and whole plant medicines were used more frequently. These conditions may be the result of screening by local residents combined with local plant resources and traditional ethnoveterinary practices.

### Methods of ethnoveterinary medicine preparation and administration

Different methods were used to prepare medicinal plants for treating livestock diseases. The most commonly used method for preparing medicinal plants was decoction (52.63%), followed by mashing (23.16%), grinding into a powder (11.58%), soaking in liquor (5.26%), and soaking in boiling water (3.16%). A few of the preparations used honey, sugar, and rapeseed oil (Table 3).

**Table 3:** Preparation methods of medicinal plants

Method of preparation	Frequency	Percentage (%)
Decoction	50	52.63
Crushing	22	23.16
Powdered	11	11.58
Medicinal liquor	5	5.26
Soak in boiling water	3	3.16
Others (add honey, oil, and sugar)	4	4.21

Medicine administration involved two modes: oral administration (64, 71.11%) and external application (26, 28.89%).

**Fig. 9:**
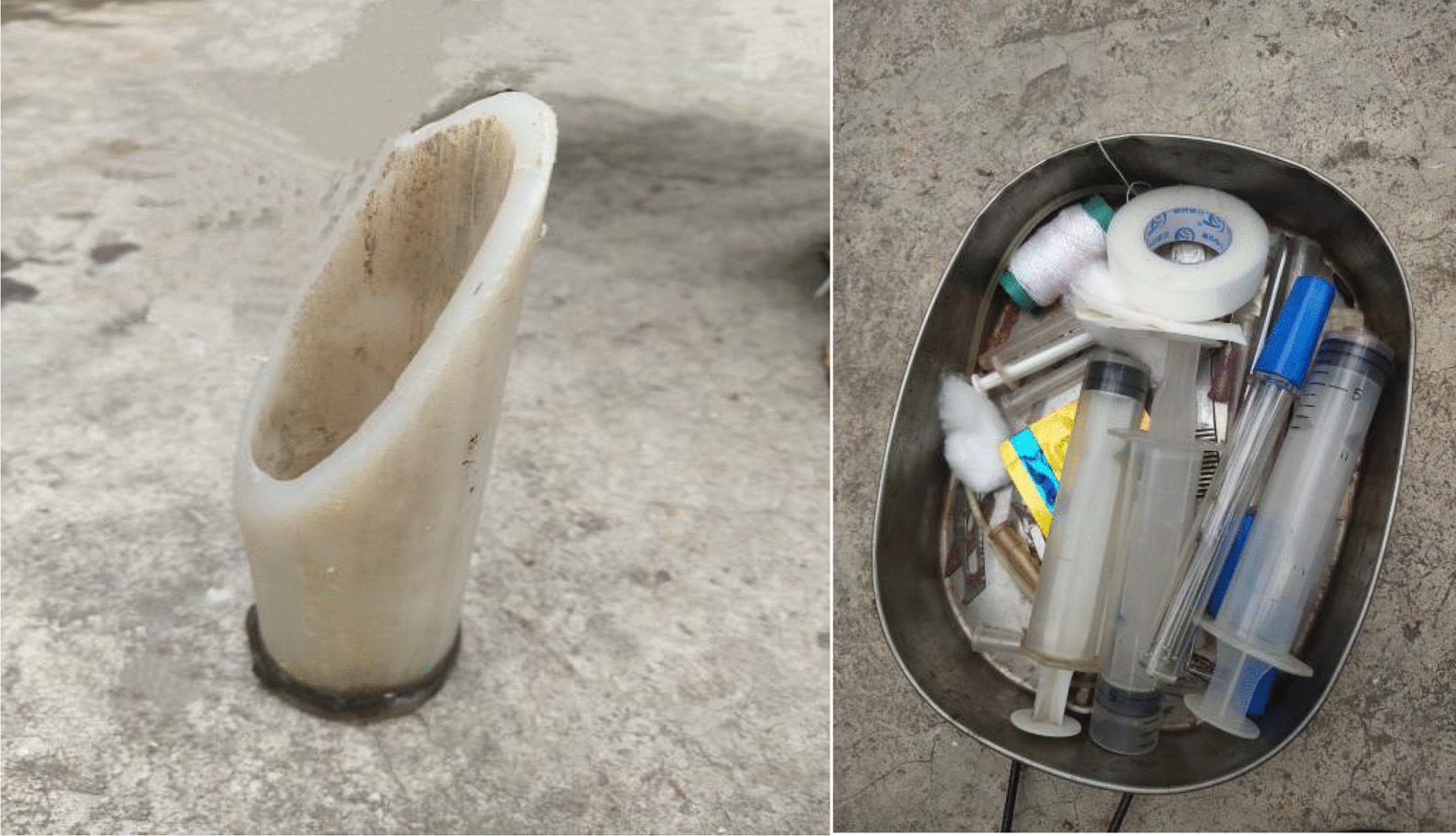
Auxiliary tools used by local veterinarians in Yunlong County was taken by Hongli Gao in Xiaomaidi on August 21, 2021

Local veterinarians used auxiliary tools for livestock that posed problems with orally administered medicines; the tools were borrowed from a local veterinary station if required. Most auxiliary tools were modified using common materials and the common auxiliary tools included feeding tools, syringes, and steel needles (Fig. 9).

## Discussion

### Characteristics of informants and their sources of traditional ethnoveterinary knowledge

Most local veterinarians and herbalists worked part-time, mainly in farming or other jobs, such as driver. They were localities with several years of experience in treating livestock diseases and most had low primary school-level education. Traditionally, in Bai culture, women are responsible for housework and men are the breadwinners of the family. Accordingly, men are responsible for feeding the livestock. Traditional ethnoveterinary practices are mainly passed on from older herbalists to the next male heir or protege. During our investigation, a 79-year-old local herbalist accepted a 40-year-old truck driver from the same village as an apprentice in Tuanjie Town. Truck drivers need to use veterinary knowledge to treat livestock in the process of transportation, and first aid experience enables truck drivers to further accumulate veterinary drug knowledge.

The traditional medical knowledge of herbal veterinarians comes from self-study or the learning practices of older generations. They continue accumulating experience in treating diseases and learning about the pharmacological effects of plants in their lifetime, which are passed down from generation to generation. In general, nobility is a requirement of the Bai community to become a healer. Many local Bai healers treat their patients without expecting anything in return.

In the past, localities who were generally poor did not charge for the treatment of other people’s livestock. This spirit of self-sacrifice in diagnosis and treatment is influenced by Buddhism and legendary tales, and the Legend of the Great Black God, the Legend of the King of Medicine, and the glazed Beast are all myths and legends about “self-sacrifice culture” [42, 43]. The “self-sacrifice culture” forms the basis of humanistic care of Bai medicine and is evident throughout the medical history of the Bai people.

### Characteristics of Bai Ethnoveterinary medicinal plants in Yunlong County

During the investigation, 90 species of medicinal plants belonging to 51 families and 84 genera were recorded and used to treat livestock diseases. Plants from the Asteraceae family were most widely used by local healers. This may be related to local hot and humid climatic conditions. Plants of the Asteraceae family, one of the largest families of seed plants worldwide, grew readily in local communities. The biomass and population size of Asteraceae e plants are typically extremely large. The Asteraceae medicinal plants are characterized by their heat-clearing and detoxifying, wind dispelling, and dehumidifying antimicrobial properties [44–46]. The medicinal plants of Asteraceae, Fabaceae, Apiaceae, Ranunculaceae, Euphorbiaceae, Gentianaceae, Lamiaceae, Polygonaceae, and Rosaceae were widely used by the localities, which may be owing to the abundance of wild plant resources in Yunlong County. This is consistent with the results of Yunfang’s investigative study on the diversity of medicinal plant resources and the dominant plant family in Yunlong County [47]. The Asteraceae and Fabaceae plants were used the most, and the results were similar to the survey results of many other research areas in southern China [48, 49].

Among 33 plant families, only one medicinal plant species was eligible as an EMP. Medicinal plants are abundant in Bai village, and local residents collect diverse medicinal species. Most EMPs are collected from wild habitats; they are dug up from near the mountains and planted in their courtyard or in the front and back of the house. In our study, *Solanum violaceum* Ortega and *Phedimus aizoon* (L.) 't Hart were planted in the courtyard of a farmer’s house, after boiling, and were fed the livestock to clear away heat and detoxify. The life forms of herbs planted in the courtyard are mostly herbs. These are regularly cared for until required during emergencies and also serve to protect endangered medicinal plants [50].

Our investigation indicated that herbal veterinarians usually went to various parts of the county to collect the necessary medicinal materials in August, thus avoiding the busy agricultural season and ensuring optimal plant growth.

Most of the harvested medicinal plants were herbs. This is not only herbs are the most used plant part for medicine, but also because they are easy to procure [49, 51]. The roots and rhizomes were the most commonly used parts for medicines, followed by whole grass, and the result is the same as other ethnic groups (Buyi, Yao, Zhuan, and Maonan) in the choice of medicinal parts [52–56]. However, this traditional utilization method causes significant damage to the biodiversity of the medicinal plants.

The selection of medicinal parts should be modified to ensure sustainable utilization of medicinal plant resources. Therefore, the resource utilization rate should be improved. The Bais have herbal medicine markets in various townships in Yunlong County. Raw herbs are used to prevent and treat various diseases. The local herbal medicinal market enriches the diversity of medicinal plants and is an important place for the exchange and dissemination of Bai medicinal culture [57]. As the education level of the older generation of herbal veterinarians is generally low, their traditional knowledge is derived from previous experiences and daily practice.

**Fig. 10:**
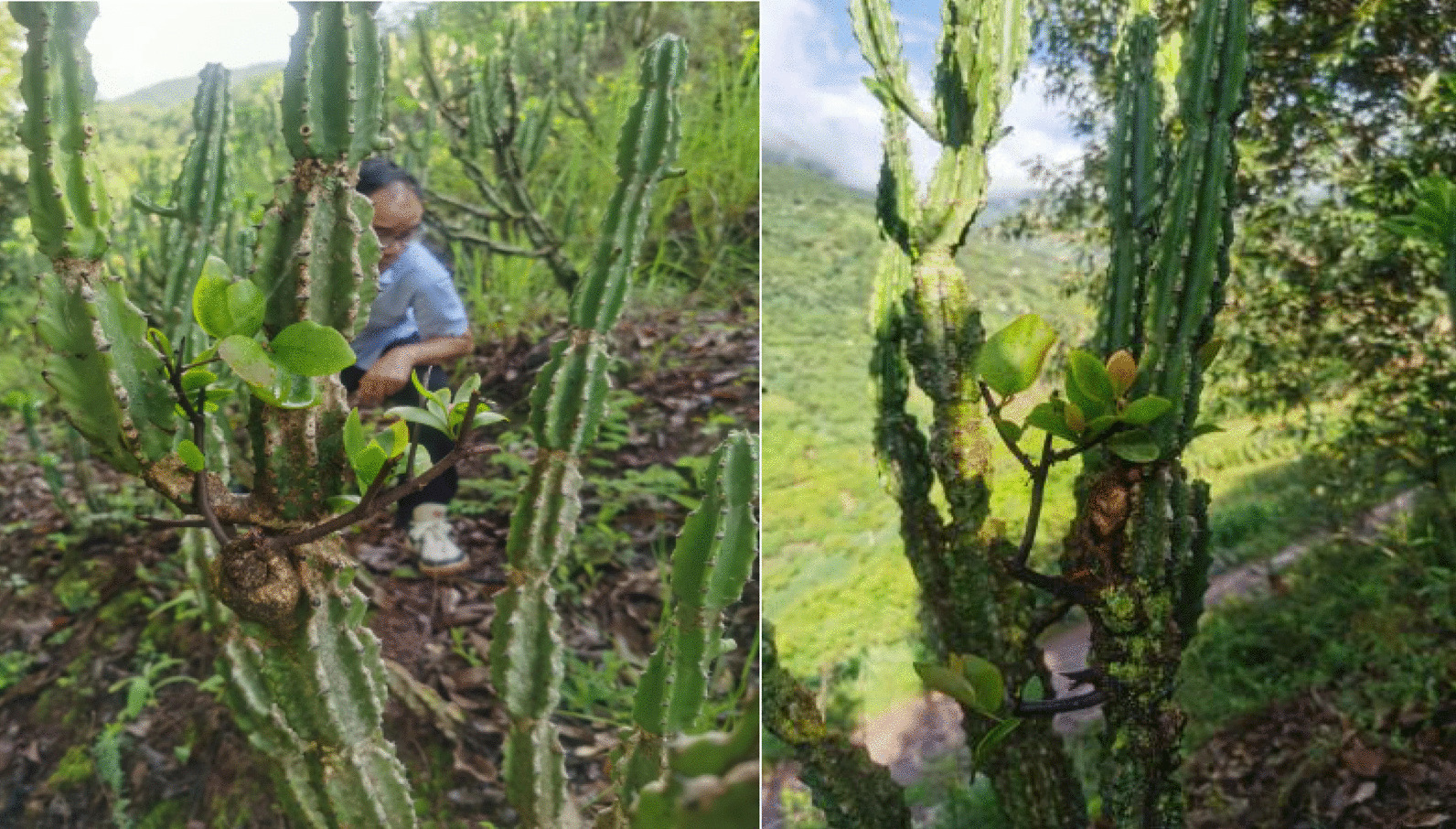
The old herbalist grafted mistletoe on the whip of *Euphorbia royleana,* was taken by Hongli Gao in Dalishu Village, Baofeng Township on July 20, 2022

Local herbalists are avant-garde and dare to accept and try new things. In our study, we encountered an old herbalist who grafted mistletoe (*Viscum coloratum*) onto a succulent plant (*Euphorbia royleana*) to improve plant growth (Fig. 10). He was able to acquire the medicinal plant *Viscum coloratum* by grafting it on the succulent plant near his home.

### Livestock breed management and treatment of livestock diseases

Outbreaks of livestock diseases seriously affect the development of aquaculture and the economic income of residents [58, 59]. Livestock breed selection is closely related to disease prevention and economic benefits. According to the interviews, veterinary staff are aware that an improvement in people’s living standards has increased the demand for meat, which the local old breed of beef cattle has been unable to meet. In 1988, local animal husbandry and veterinary management departments began to cross-breed old cattle breeds free of charge to increase the number of beef cattle. The hybrid cattle were strong, disease-resistant, and highly valued. Cross-breeding is now mostly performed by veterinary station staff, which charges 100–300 RMB each time. As older yellow cattle breed are small and rarely get sick, they are more suitable for free breeding in the local mountainous environment; therefore, there is still a large stock in the Yunlong Bai region.

Local veterinarians diagnose livestock diseases based on existing medical knowledge. Common diagnostic methods include observation (e.g., observing the physical manifestations and disease symptoms), listening and smelling (e.g., listening to the sound and breath of animals, sniffing secretions, and excreta), questioning (e.g., asking animal keepers about the appearance or history of the disease), and palpation (e.g., touching or pressing the animal’s body, feeling the pulse, and other viscera), which is similar to traditional Chinese medicine [60, 61]. Tongue examination is not only an important part of traditional Bai medicine but also an important component of disease diagnosis [62].

Red fur is wind-cold, yellow fur indicates excess heat, green fur indicates toxicosis, and white fur indicates collapse. After diagnosing the disease, the local veterinarians begin to prescribe the right medicine for the case. Suitable medicinal plants can be used to prevent and treat diseases owing to their medicinal properties.

*Rodgersia sambucifolia* is widely used by local veterinarians for the treatment of livestock respiratory diseases owing to the plant’s polyphenols, flavonoids, terpenoids, and volatile oils [63, 64]. Farmers and herbal veterinarians use varied methods to treat their livestock. They often mash* Selaginella moellendorffii *and feed it to the animals to treat a postpartum abdominal cold for livestock postnatal care. Because of the hot and humid climate in Yunlong County, the Asteraceae medicinal plants (*Chrysanthemum indicum* L., *Taraxacum mongolicum* Hand.-Mazz., *Aucklandia costus* Falc., etc.) are often mashed or boiled and fed to livestock for heat-clearing and detoxification.

In addition to using plants to prevent livestock diseases, local veterinarians have developed unique diagnostic and treatment methods. They use gunpowder to wipe the fur of livestock to treat depilation and apply gasoline to the wounds to ward off maggots.

The donkey turned mad and pulled up the long mane on top of its head and put it in with a needle supplemented with cat incense (wildcat secretion) internal administration can cure. The tripe flatulence was inserted directly with a steel needle, turned down, and taken internally with *Rodgersia sambucifolia* Hemsl, *Actaea cimicifuga* L., and other medications after bloodletting and outgassing.

### Prospects and challenges of traditional ethnoveterinary knowledge

Although Chinese traditional medical theory is famous worldwide for its application in human health, it is rarely mentioned in countries other than China. Traditional Chinese medicine has been used in veterinary medicine and human medicine practice in China for thousands of years.

In modern Chinese society, herbs used for the treatment of animal diseases or animal feed are believed to contain fewer residues than traditional medicines [65]; moreover, they are believed to reduce bacterial drug resistance and food safety problems caused by modern veterinary drugs [66]. Notices numbers 194 and 246 of the Ministry of Agriculture and Villages of the People’s Republic of China have led to the ban of the addition of antibiotics to veterinary drugs. Conversely, the various standards of traditional Chinese medicine allowing feed additives for both growth promotion and prevention and control have been revised [67, 68].

Therefore, EMPs will gradually be welcomed in the prevention and control of diseases and the health protection of livestock. In remote and poor areas, EMPs are the first choice for local prevention and treatment of livestock diseases. However, under the influence of the mainstream social economy, an increasing number of people choose to work in cities, which hinders the inheritance of the traditional medicine culture and decreases the traditional animal husbandry and the number of animals in rural areas. In this survey, a number of practicing veterinarians said that children in their families would rather sell tea or work in a factory than learn about veterinary medicine.

Currently, most people with knowledge of traditional medicinal plants and their use are over 50 years of age. They mostly engage in agricultural labor or breeding and rely only on their spare time to acquire traditional veterinary medical knowledge. These results threaten the inheritance of EMPs and traditional medical knowledge.

## Conclusion

Traditional veterinary medicine is easy to master and perform and is inexpensive. It plays an important role in the development of local aquaculture and animal husbandry and is the first choice for the prevention and treatment of animal diseases in remote and poor areas.

However, with the passing on of the older generation, traditional knowledge of EMPs may disappear. In this study, we collected and sorted traditional knowledge about medicinal plants used in veterinary practice in Yunlong County. We obtained information on 90 EMPs and their corresponding treatment types for livestock diseases and studied the life form, drug preparation, and mode of administration of EMPs. This study plays an important role in the protection and inheritance of Bai EMPs and their traditional knowledge in Yunlong County.

Traditional knowledge of ethnoveterinary medicine is related to the local social–cultural characteristics of the Bai people and plays a pivotal role in livestock production. Plants are the carriers of traditional culture, and traditional culture nourishes plant culture. Cultural diversity and biodiversity depend on each other. The traditional community has extremely rich traditional knowledge related to the improvement of people’s health and environmental hygiene conditions.

### Supplementary Information

**Additional file 1: Table S1.** Plants and their used in ethnoveterinary medicine by Bai people.

## Data Availability

Not applicable.

## Abbreviations

FIC: The informant consensus factor
URs: Use reports
EMPs: Ethnoveterinary medicine plants
