# Supplementary material for: An ethnoveterinary study on medicinal plants used by the Bai people in Yunlong County northwest Yunnan, China
Source: J Ethnobiol Ethnomed. 2024 Jan 13;20:9. doi: 10.1186/s13002-023-00633-0 (PMC10787986; doi:10.1186/s13002-023-00633-0)
Supplement: Supplementary file 1 — Additional file 1: Table S1. Plants and their used in ethnoveterinary medicine by Bai people. [file 13002_2023_633_MOESM1_ESM.docx]

| Table S1 Plants and their used in ethnoveterinary medicine by Bai people | | | | | | |  |
| --- | --- | --- | --- | --- | --- | --- | --- |
| Voucher number | Scientific name | Family name | Vernacula name | Habit | Use part | Medicinal uses | URs |
| Ghl01 | *Angelica dahurica* (Hoffm.) Benth. & Hook.f. ex Franch. & Sav. | Apiaceae | 嗯防风  nged fanpfenx | Herb | Root | Dry of the herb are crushed and mixed with [borneol](javascript:;), and it is coated to livestock for exterior inflamed skin. | 35 |
| Ghl02 | *Actaea cimicifuga* L. | Ranunculaceae | 钱生嘛  qiaidsenxmap | Herb | Rhizome | Dry roots are boiled and orally given to livestock for treatment of throat. | 42 |
| Ghl03 | *Agastache rugosa*  (Fisch. & C.A.Mey.) Kuntze | Lamiaceae | 活香  hufxianx | Herb | Branch  Leaf | A whole plant is boiled and orally given to  livestock for treatment of sunstroke. | 43 |
| Ghl04 | *Artemisia argyi* H.Lév. & Vaniot | Asteraceae | 瀚  hanl | Herb | Leaf | Fresh leaves are taken and crushed, then mixed with fodder and orally given to pig for treatment of [threatened abortion](javascript:;). | 51 |
| Ghl05 | *Achyranthes aspera* L. | Amaranthaceae | 次瓜子  cilgua zi | Herb | Root | A whole plant is boiled and orally given to  livestock for treatment of retained placenta. | 32 |
| Ghl06 | *Astragalus yunnanensis* Franch. | Fabaceae | 黄琴  huanpqinx | Herb | Root | Powder of its roots is orally given to livestock as tonic | 9 |
| Ghl07 | *Artemisia annua* L. | Asteraceae | 干汉子  ganlhanlzi | Herb | Stem  Leaf | Smash and warm boiled water to clear heat and relieve summer heat. | 49 |
| Ghl08 | *Argentina stenophylla* (Franch.) Soják | Rosaceae | 图炉子  tuxlulzi | Herb | Whole | Fresh root of the herb is crushed and made  to paste and coated afflicted part to treat traumatic injury. | 25 |
| Ghl09 | *Arctium lappa* L. | Asteraceae | 羊棒子  yanxbanbzi | Herb | Root | Decortion is made from its seed, and orally given to ruminant for treatment of obstructed milk. | 37 |
| Ghl10 | *Botrychium ternatum* (Thunb.) Sw. | Ophioglossaceae | 费心草  feibxinxcaox | Herb | Whole | The whole plant is boiled and orally given to livestock for treatment of [pneumonia](javascript:;). | 50 |
| Ghl11 | *Boenninghausenia albiflora* (Hook.) Rchb. ex Meisn. | Rutaceae | 白虎草  baifhutcux | Herb | Whole | Fresh herb are crushed and made rake to cattle for treatment of [traumatic injury](javascript:;). | 11 |
| Ghl12 | *Berberis diaphana* Maxim. | Berberidaceae | 给够起  geilgo qix | Shrub | Root  Stem | Decoction is made from its roots, and orally given to livestock for treatment of diarrhea | 47 |
| Ghl13 | *Berberis fortunei* Lindl. | Berberidaceae | 十大功劳  sildabgonxlaop | Shrub | Root  Stem | Decoction is made from its root, and orally given to piglet for treatment of diarrhea. | 41 |
| Ghl14 | *Bupleurum scorzonerifolium* Willd. | Apiaceae | 柴胡  caiphux | Herb | Root | Decoction is made from its roots, and orally given to livestock for treatment of cold and fever. | 47 |
| Ghl15 | *Begonia grandis* Dryand. | Begoniaceae | 八旭龙  bafxufnop | Herb | Root | Fresh root of the herb is crushed and made to paste and coated afflicted part for treat traumatic injury. | 32 |
| *Ghl16* | *Conioselinum smithii* (H.Wolff) Pimenov & Kljuykov | Apiaceae | 去凶  quilxionl | Herb | Root | Decoction is made from its root, and orally given to livestock for treatment of  blood stasis. | 45 |
| Ghl17 | *Clematis chinensis* Osbeck | Ranunculaceae | 威灵仙  weixlinpxian | Climber | Root | Decoction is made from its bulb, and orally given to cattle for treatment of throat trouble. | 35 |
| Ghl18 | *Cymbopogon citratus* (DC.) Stapf | Poaceae | 云香草  yunpxianxcaoc | Herb | Stem  Leaf | Steams and leaves are boiled and orally given to livestock for treatment of appetite. | 30 |
| Ghl19 | *Celosia argentea* L. | Amaranthaceae | 气凶  qilxionl | Herb | Seed | It’s seed is combined with fodder, given orally to livestock for heat-clearing and detoxifying. | 15 |
| Ghl20 | *Coptis chinensis* Franch. | Ranunculaceae | 喔百起  ngotbaiqit | Herb | Rhizome | Powder of its roots is orally given to piglet for treatment [diarrhea](javascript:;). | 45 |
| Ghl21 | *Chrysanthemum indicum* L. | Asteraceae | 及枯后  jilkv hol | Herb | Flowers | Root is boiled and orally given to horse for treatment of urinary trouble. | 12 |
| Ghl22 | *Commelina communis* L. | Commelinaceae | 阿在厨  atze cux | Herb | Whole | The whole plant is boiled and orally given to livestock for treatment of throat trouble. | 10 |
| Ghl23 | *Curculigo capitulata* (Lour.) Kuntze | Hypoxidaceae | 几宗  jitzonl | Herb | Rhizome | Root is boiled and orally given to livestock as tonic. | 15 |
| Ghl24 | *Dolomiaea costus* (Falc.) Kasana & A.K.Pandey | Asteraceae | 木香蜜  mofxianxmip | Herb | Root | Decoction is made from its root, and orally given to livestock for invigorating stomach. | 38 |
| Ghl25 | *Disporopsis fuscopicta* Hance | Asparagaceae | 竹跟七  zufgenxqil | Herb | Rhizome | Smash and apply the afflicted part , activate blood circulation and remove blood stasis. | 15 |
| Ghl26 | *Davallia trichomanoides* Blume | Polypodiaceae | 挂得补  guadetbut | Herb | Rhizome | Fresh roots are taken and crushed and made rake to horse ([mule](javascript:;)) for treatment of fracture. | 10 |
| Ghl27 | *Dactylicapnos scandens* (D.Don) Hutch. | Papaveraceae | 白起  bai qi | Climber | Root | Root is crushed and made o paste and coated afflicted part for treatment of traumatic injury. | 19 |
| Ghl28 | *Duhaldea cappa* (Buchanan-Hamilton ex D. Don) Pruski & Anderberg | Asteraceae | 勇野得挂  yondyinxdetgual | Shrub | Whole | The whole plant is boiled and orally given to livestock for treatment of stomachache. | 15 |
| Ghl29 | *Euphorbia pekinensis* Rupr. | Euphorbiaceae | 逗单色  dopdanlse | Herb | Root | Fresh root of the herb is crushed and made to paste and coated afflicted part to smash and apply externally, reduce swelling and disperse knots. | 30 |
| Ghl30 | *Eucommia ulmoides* Oliv. | Eucommiaceae | 肚重  dubzob | Tree | Peel | Mash and apply externally to strengthen muscles and bones | 49 |
| Ghl31 | *Euphorbia lathyris* L. | Euphorbiaceae | 地搞子  dibgaotzi | Herb | Seed | Decoction is made from its seed, and orally given to ruminant for treatment of impaction of rumen.. | 6 |
| Ghl32 | *Eupatorium fortunei* Turcz. | Asteraceae | 香草  xiolcux | Herb | Whole | Powder of its roots is orally given to piglet for treatment gastric disorder. | 40 |
| Ghl33 | *Euphorbia helioscopia* L. | Euphorbiaceae | 五堵云  wuxduxyunp | Herb | Whole | The whole plant is crushed and made o paste and coated afflicted part for treat ringworm ulcer. | 15 |
| Ghl34 | *Fagopyrum cymosum* (Trevir.) Meisn. | Polygonaceae | 及跟谷  jilgelgud | Herb | Tuber  root | Along with *Houttuynia cordata*, fresh roots are boiled and orally given to livestock for prevention the cough. | 14 |
| Ghl35 | *Gentiana crassicaulis*  Duthie ex Burkill | Gentianaceae | 琴啾  qinxjiox | Herb | Root | Root is boiled and orally given to horse for treatment of pneumonia. | 42 |
| Ghl36 | *Glycyrrhiza uralensis* Fisch. ex DC. | Fabaceae | 杠草  ganlcaot | Herb | Root  Rhizome | Decoction is made from its roots, and orally given to cattle for treatment of gaseous distention. | 48 |
| Ghl37 | *Geranium nepalense* Sweet | Geraniaceae | 老霍草  laothufcaox | Herb | fruit | Decoction is made from its aboveground parts with fruit, and orally given to cattle for treatment of enteritis. | 8 |
| Ghl38 | *Gynostemma pentaphyllum* (Thunb.) Makino | Cucurbitaceae | 比噗子  bilpolzi | Herb | Whole | Fresh leaves are taken and crushed, and after that, are combined with fodder, given orally to livestock for treatment of [chronic gastroenteritis](javascript:;). | 21 |
| Ghl39 | *Gynura japonica* (Thunb.) Juel. | Asteraceae | 猜弄  caitnol | Herb | Root | Grind into powder and apply to the affected area to dissipate blood stasis and stop bleeding. | 9 |
| Ghl40 | *Gardenia jasminoides* J.Ellis | Rubiaceae | 子子  zilzi | Shrub | Fruit | Steam the fruit and is orally given to piglet for treatment of icteric . | 7 |
| Ghl41 | *Gentiana scabra* Bunge | Gentianaceae | 色枯草  sefkuxcaot | Herb | Root  Rhizome | Root is boiled and orally given to livestock for treatment of [hepatitis](javascript:;). | 32 |
| Ghl42 | *Hypericum uralum* Buch.  -Ham. ex D.Don | Hypericaceae | 寿走厚  solzodhol | Shrub | Root | Decoction is made from its root, and orally given to livestock for treatment of upper respiratory tract infection. | 45 |
| Ghl43 | *Houttuynia cordata* Thunb. | Saururaceae | 虾厨蜜  xiailcuxmi | Herb | Whole | A whole plant is boiled and orally given to piglet for treatment of diarrhea . | 19 |
| Ghl44 | *Humulus scandens* (Lour.) Merr. | Cannabaceae | 律草  lufcaot | Herb | Whole | The whole plant is boiled and orally given to livestock for treatment of diuretic swelling. | 5 |
| Ghl45 | *Lonicera japonica* Thunb*.* | Caprifoliaceae | 戈厨  geilcvnt | Herb | Flowers | Decoction is made from its dry flowers, and orally given to cattle for treatment of cold. of nosebleed. | 24 |
| Ghl46 | *Ligularia sibirica* (L.) Cass. | Asteraceae | 脂韵  zixyunb | Herb | Root  Rhizome | Decoction is made from its roots, and orally given to livestock for treatment of cough and asthma. | 7 |
| Ghl47 | *Lycopodium japonicum* Thunb. | Lycopodiaceae | 宽金草  kuaixjinxcaot | Herb | Whole | A whole plant is boiled and scrubs the afflicted part to treat the injury. | 51 |
| Ghl48 | *Lithospermum erythrorhizon* Siebold & Zucc. | Boraginaceae | 泽当  celdanx | Herb | Root | Root is boiled and orally given to livestock for treatment of urinary trouble. | 20 |
| Ghl49 | *Leontopodium leontopodinum* (DC.) Hand.-Mazz. | Asteraceae | 翻白菜  fanxbaicet | Herb | Whole | The whole plant is boiled and orally given to livestock for treatment of moistening the lungs and relieving cough. | 16 |
| Ghl50 | *Mentha canadensis* L. | Lamiaceae | 宝虎  baothot | Herb | Whole | Decoction is made from its bulb, and orally given to livestock for treatment of intestinal impaction. | 21 |
| Ghl51 | *Melia azedarach* L. | Meliaceae | 户腥子  hulxinlzi | Tree | Peel | Powder of its peel is boiled and orally given for treatment of the removal worms. | 39 |
| Ghl52 | *Ophiopogon japonicus* (Thunb.) Ker Gawl. | Asparagaceae | 濹东  mefdonx | Herb | Tuber  root | Powder of its roots is mixed with fodder, then orally given to livestock for treatment bronchitis. | 12 |
| Ghl53 | *Paris polyphylla* Sm. | Melanthiaceae | 浓摸噗  nodmoxpul | Herb | Root  Stem | Decoction is made from its roots, and then, it is combined with fodder, given orally to livestock for heat-clearing and detoxifying. | 50 |
| Ghl54 | *Platycladus orientalis* (L.) Franco | Cupressaceae | 宫凶百  gonpxiolbai | Tree | Leaf  Branch | Decoction is made from its leaves, and orally given to cattle for treatment of nosebleed. Powder of its roots is orally given to livestock for treatment of traumatic injury. | 25 |
| Ghl55 | *Pueraria edulis*  Pamp*.* | Fabaceae | 苏沫  sulmol | Climber | Root | Decoction is made from its roots, and orally given to cattle for treatmentof fever. | 25 |
| Ghl56 | *Plantago asiatica* L. | Plantaginaceae | 得母干那色  deipmoxganfnanlse | Herb | Whole | The whole plant is boiled and orally given to livestock for treatment of urinary tract infections. | 45 |
| Ghl57 | *Phedimus aizoon* (L.) 't Hart | Crassulaceae |  | Herb | Whole | The whole herb is taken and crushed, and scrubs the afflicted part to livestock for treatment of mosquito bite. | TH |
| Ghl58 | *Paederia foetida* L. | Rubiaceae | 给史蜜  geilsixmi | Climber | Vine | Fresh tender leaves are crushed and applied externally to treat traumatic stasis. | 16 |
| Ghl59 | *Pinellia ternata* (Thunb.) Makino | Araceae | 半下  baibxiab | Herb | Tuber | Powder of its roots is boiled and orally given for the treatment of stomach Problems. | 15 |
| Ghl60 | *Portulaca oleracea* L. | Portulacaceae | 买子巴子  maixzi bapzi | Herb | Whole | Fresh herb are taken and crushed and made rake to cattle for treatment of fracture. | 12 |
| Ghl61 | Phryma leptostachya L. | Phrymaceae | 透谷草  tobgufcaot | Herb | Root | Fresh roots are taken and crushed, then add a small amount of salt to mash and apply externally to carbuncle and swelling. | 7 |
| Ghl62 | *Polygala arillata* Buch.-Ham. ex D.Don | Polygalaceae | 金雀花  jinxqufhuax | Shrub | Root | Root is boiled and orally given to livestock for treatment of [hepatitis](javascript:;). | 31 |
| Ghl63 | *Polygonatum sibiricum* Redouté | Asparagaceae | 酸色子  suanlse zi | Herb | Rhizome | Powder of its roots is mixed with fodder, then orally given to livestock for treatment of pulmonary disease. | 39 |
| Ghl64 | *Platycodon grandiflorus* (Jacq.) A.DC. | Campanulaceae | 及埂  jilgenx | Herb | Root | Decoction is made from its roots, and orally given to remove phlegm and relieve cough. | 18 |
| Ghl65 | *Prunella vulgaris* L. | Lamiaceae | 梅追厨  meidzulcut | Herb | Whole | A whole plant is boiled and orally given to  livestock for treatment of icterus. | 24 |
| Ghl66 | *Polygonatum sibiricum* Redouté | Rosaceae | 枯把子  kuxba zi | Herb | Whole | The whole herbs is crushed and made o paste and coated afflicted part for treat for venomous snake bites. | 45 |
| Ghl67 | *Potentilla discolor* Bunge | Rosaceae | 繁白一  fanxbaifyif | Herb | Whole | The whole plant is boiled and orally given to livestock for treatment of dysentery. | 32 |
| Ghl68 | *Pentanema britannica* (L.) D.Gut.Larr., Santos-Vicente, Anderb., E.Rico & M.M.Mart.Ort. | Asteraceae |  | Herb | Whole | Fresh root of the herb is crushed and made  to paste and coated afflicted part for relieve swelling and pain. | 35 |
| Ghl69 | *Reynoutria japonica* Houtt. | Polygonaceae | 换拖  huainl tux | Herb | Root  Stem | A whole plant is boiled and orally given to livestock for treatment of traumatic injury. | 28 |
| Ghl70 | *Rhodiola yunnanensis* (Franch.) S.H. Fu | Crassulaceae | 给志弄  geilzilnol | Herb | Whole | Decoction is made from its roots and orally administered to cattle for the treatment of diarrhea relieve cough and asthma | 18 |
| Ghl71 | *Rodgersia sambucifolia* Hemsl. | Saxifragaceae | 啥哟  ssapyo | Herb | Rhizome | Decoction is made from its roots and orally  administered to pig for the treatment of diarrhea | 59 |
| Ghl72 | *Rheum palmatum* L. | Polygonaceae | 土大黄  tutdabhuanp | Herb | Root  Rhizome | Decoction is made from its bulb, and orally given to cattle for treatment of [constipation](javascript:;). | 5 |
| Ghl73 | *Schisandra rubriflora* Rehder & E.H.Wilson | Schisandraceae | 濹以子  me yit zi | Climber | Fruit | Fruits of the herb are fried，then mixed with fodder and orally given to horse for treatment of cough. | 38 |
| Ghl74 | *Selaginella moellendorffii* Hieron. | Selaginellaceae | 卷白  juitbaif | Herb | Whole | Fresh root of the herb is crushed and made  to paste and coated afflicted part to cattle for treatment arthritis | 53 |
| Ghl75 | *Solanum violaceum* Ortega | Solanaceae | 夸固子  kuai gulzi | Shrub | Root  Leaf  Fruit | The whole herb is taken and crushed, and scrubs the apply to the skin to repel parasites. | 21 |
| Ghl76 | *Stellera chamaejasme* L. | Thymelaeaceae | 首山火  soxsanxhux | Herb | Root | Root is soaked in liquor, and wipe the afflicted part to reduce inflammation and relieve pain. | 16 |
| Ghl77 | *Solanum erianthum* D.Don | Solanaceae | 斗嘛色  dopmadse | Tree or Shrub | Whole | Fresh root of the herb is crushed and made  to paste and coated afflicted part to treat traumatic injury. | 18 |
| Ghl78 | *Saposhnikovia divaricata* (Turcz. ex Ledeb.) Schischk. | Apiaceae | 酒史得  jionpsitde | Herb | Root | The whole plant is boiled and orally given to livestock for treatment of cold. | 42 |
| Ghl79 | *Sagittaria trifolia* L. | Alismataceae | 古得子  guldetzi | Herb | Root | Decoction is made from its roots, and orally given to livestock for treatment of dispersing knots and reducing swelling. | 15 |
| Ghl80 | *Saxifraga stolonifera* Curtis | Saxifragaceae | 篓烟得  laodyanxde | Herb | Whole | Decoction is made from the whole herb, and orally given to livestock for treatment of fever. | 19 |
| Ghl81 | *Swertia leducii* Franch. | Gentianaceae | 须白扯  xuxbaicet | Herb | Whole | The whole plant is boiled and orally given to livestock for treatment of hepatitis. | 41 |
| Ghl82 | *Taxillus sutchuenensis*  (Lecomte) Danser | Loranthaceae | 桑记森  sanxjibsenx | Shrub | Branch  Leaf | Dry of the branch with leaf are crushed. these are boiled and orally administered to cattle for treatment of miscarriage prevention. | 36 |
| Ghl83 | *Tamarix ramosissima* Ledeb. | Tamaricaceae | 桂柳  guiphhex | Tree | Branch  Leaf | Decoction is made from its leaves, and orally given to livestock for treatment of bronchitis. | 9 |
| Ghl84 | *Taraxacum mongolicum* Hand.-Mazz. | Asteraceae | 巴齿色  balcitsei | Herb | Root | Dry of the herb are crushed and mixed with fodder for anti-bacteria. | 47 |
| Ghl85 | *Torricellia angulata* Oliv. | Torricelliaceae | 甲刮又  jia gua yo | Tree | Leaf | Fresh leaves are taken and crushed and made rake to cattle for treatment of fracture. | 75 |
| Ghl86 | *Valeriana jatamansi* Jones ex Roxb. | Caprifoliaceae | 兄包子  xiolbaol z | Herb | Root | Root is boiled and orally given to horse for blood circulation and reduce swelling. | 40 |
| Ghl87 | *Viola philippica* Cav. | Violaceae | 及白云子  ji baipngvlzi | Herb | Whole | Mash the fresh herb and apply it to the treatment of suppurative infection of cattle. | 8 |
| Ghl88 | *Wisteria sinensis* (Sims) DC. | Fabaceae | 格刷蜜  geilsua mi | Climber | Seed | Power of seed is sprinkled on the afflicted part to remove worm. | 10 |
| Ghl89 | *Zingiber officinale* Roscoe | Zingiberaceae | 贡  gonl | Herb | Rhizome | Fresh ginger is washed and broken to extract juice, which is fed to livestock to promote diuresis and detoxification. | 65 |
| Ghl90 | *Zea mays* L. | Poaceae | 卢古  luxgut | Herb | Corn  whisker | Decoction is made from its fresh stigmata maydis, and orally given to ruminant for treatment of icterus. | 15 |
